# Metal-Enhanced Fluorescence of Nanocomplexes

**DOI:** 10.3390/ma19061258

**Published:** 2026-03-22

**Authors:** Alexander N. Yakunin, Sergey V. Zarkov, Yuri A. Avetisyan, Garif G. Akchurin, Valery V. Tuchin

**Affiliations:** 1 Laboratory of Laser Diagnostics of Technical and Living Systems, IPMC RAS, FRC “Saratov Scientific Centre of the RAS”, 410028 Saratov, Russiayuaavetisyan@mail.ru (Y.A.A.); akchuringg@mail.ru (G.G.A.); tuchinvv@mail.ru (V.V.T.); 2 Institute of Physics and Science Medical Center, Saratov State University, 410012 Saratov, Russia; 3 Laboratory of Biophotonics, Tomsk State University, 634050 Tomsk, Russia

**Keywords:** metal-enhanced fluorescence, fluorescence nanocomplexes, spectral properties of fluorophore molecule, fluorescence spectral response

## Abstract

Metal-enhanced fluorescence (MEF) has found widespread application in biomedical sensing and in vivo tissue imaging systems. To enhance MEF efficiency, it is necessary to optimize the interaction between the metal nanoparticle plasmon and the fluorophore molecule. The size and shape of the nanoparticle, the nanoscale gap between the fluorescent molecule and the nanoparticle, and the excitation wavelength are critical parameters. In this study, we propose a model for a more complete and accurate description of the processes of molecular excitation and generation of the fluorescence spectral response, introducing a new concept of effective properties for the field enhancement factor, quantum yield, and fluorescence enhancement factor. The influence of the spectral properties of both the nanostructure plasmon and the fluorophore molecule on the optimal tuning of fluorescent complexes is studied. Particular attention is paid to the analysis of the spectral properties of plasmon resonance and calculations of the near-field intensity enhancement of the plasmonic nanostructure’s excitation field. Numerical results for optimizing the MEF of fluorescent complexes based on TagRFP and gold (silver) nanorod composites are presented. The advantages of the proposed model for the optimal design of new nanomaterials with unique fluorescent properties are discussed.

## 1. Introduction

The phenomenon of metal-enhanced fluorescence has become widespread in biomedical sensing systems [1,2], in vivo imaging of biological tissues [3,4], and detection of highly toxic pollutants resulting from natural and anthropogenic activities [5,6]. Improving MEF efficiency requires precisely tuning the plasmon resonance of a nanoparticle to ensure optimal conditions for the interaction of its plasmon with the fluorophore molecule. The controlling parameters are the size and shape of the nanoparticle [7], the size of the nanoscale gap between the molecule and the nanoparticle, and the irradiation wavelength [8,9,10,11].

The spatial resolution achieved by near-field optical technology, thanks to the MEF effect [12,13], enables the detection of individual molecules [9,10,11,14]. Single-molecule fluorescence spectroscopy is also actively developing for studying biological systems at the molecular level. These optical methods have enabled the study of protein and deoxyribonucleic acid (DNA) dynamics and facilitated the development of methods for sequencing individual DNA molecules [15,16,17].

MEF-based technologies have also found application in biomedical diagnostics, bioimaging, and therapy [18,19]. Enhancement of the fluorescence intensity of individual fluorescent protein molecules near the surface of noble metals (silver or gold) was discovered over 40 years ago [20]. Electrodynamic approaches based on the model of an emitting and absorbing point dipole as a fluorophore molecule qualitatively confirmed this phenomenon [21]. Probe atomic force microscopy technology, containing a single gold plasmonic spherical nanoparticle at the probe tip, made it possible to study the dependence of the fluorescent molecule enhancement on the distance between the nanoantenna tip and an individual fluorophore molecule, as well as to experimentally confirm the effects of fluorescence quenching and establish the corresponding dependences on the gap between the fluorophore molecule and the nanoparticle [22].

Irradiation of the complex with an external plane optical wave at a wavelength λ_ex_, corresponding to the electronic excitation energy of the fluorophore molecule, is accompanied by the formation of near-field localization zones in the vicinity of a plasmon-resonant nanoparticle made of various metals (e.g., silver and gold). A characteristic feature of fluorescence is that even with monochromatic irradiation of the fluorophore, a fluorescence response (fluorescence signal) is observed with a relatively broad spectrum with a maximum at a wavelength λ_em_, shifted relative to λ_ex_ by the Stokes shift [23]. The MEF effect of increasing the radiative velocity of emission and, accordingly, decreasing the lifetime of the fluorophore in the excited state is due to the so-called Purcell effect—an increase in the local density of photon states in the nanoresonator created by the plasmonic nanoparticle and the fluorophore molecule [23,24].

Furthermore, a competing tunneling effect occurs, causing a sharp quenching of fluorescence intensity. This occurs when the minimum distance between the nanoparticle surface and the fluorophore molecule becomes less than a few nanometers, as confirmed by probe measurements using an atomic force microscope [25]. This process of fluorescence quenching via the Forster mechanism at typical distances of less than 5 nm suppresses the fluorescence enhancement effect via the Purcell mechanism [26].

It should be noted that typical probe measurements of the fluorescence of individual fluorophore molecules containing a sufficiently large number of atoms are conducted under laboratory conditions, i.e., not under high vacuum or at liquid helium temperatures. Therefore, the fine vibrational–rotational energy structure is not experimentally observed in either the fluorophore absorption spectrum or the fluorescence spectrum of the individual emitting molecules. For numerical calculations that take into account the spectral dependences of fluorophores, it is possible to use the international experimental database for fluorescent dyes, which represent continuous spectral dependences for both the electron absorption spectrum of the fluorophore and the fluorescence spectrum [27].

The proposed strategy for optimizing the fluorescence enhancement coefficient for an individual fluorophore molecule, taking into account the actual fluorescence spectra of the molecules, should be determined by the spectral overlap of the controlled surface plasmon resonance of the nanoparticle (the position of the spectral maximum of the plasmon resonance (PR) and its spectral width) with the absorption spectrum of the fluorophore and its fluorescence spectrum.

Experimental studies have shown that the choice of the spectral maximum of the plasmon resonance between the spectral maxima of absorption and fluorescence becomes unclear at a fixed distance between the molecule and the nanoparticle, compared to tuning to the fluorophore absorption maximum or the fluorescence maximum [28]. This makes the task of spectral optimization of fluorescence enhancement nontrivial for finding a definitive solution. One of the main reasons is that the MEF mechanism is determined by two processes: the enhancement of the local optical field formed by the nanoparticle in the near zone, and affecting the local absorption of the fluorophore molecule, and the increase in the rate of spontaneous emission of the fluorescent molecule in the nanoresonator (the Purcell effect). The latter process was experimentally confirmed in a study of the dynamic response of fluorophore molecules with nanosecond resolution and was manifested in a decrease of more than an order of magnitude in the emission time of fluorophore molecules with a nanoparticle with a fixed DNA-based linker [29]. At typical distances between the surface of the plasmonic nanoparticle and the fluorescent molecule of less than 5 nm, the competing process of fluorescence quenching by the Förster mechanism is intensively activated [10].

Numerical modeling of the radiative and nonradiative relaxation rates of a fluorophore molecule demonstrates a critical dependence on the spatial nanoscale gap between the plasmonic nanoparticle and the molecule [9]. Therefore, these factors introduce significant adjustments to the conditions for achieving maximum fluorescence enhancement. Numerical electrodynamic and quantum models of fluorescence enhancement using plasmon-resonant nanoparticles have evolved from the assumption that the fluorophore absorption wavelength coincides with the emission wavelength [9] to a more realistic monochromatic model, where the fluorophore molecule transitions to an excited electronic state at a wavelength λ_ex_, corresponding to the maximum absorption of the fluorophore molecule, and emission occurs at a wavelength λ_em_, differing by the Stokes shift and corresponding to the maximum fluorescence value [29].

In this case, the controlling parameters are the position of the plasmon resonance maximum and the spectral shape of the resonance curve relative to these wavelengths λ_ex_ and λ_em_. In developing this electrodynamic approach, we believe it is necessary to consider not only the maximum values of the fluorophore absorption and emission wavelengths but also the actual spectral dependence of the absorption and fluorescence of the dye. Failure to consider the spectral dependence of the fluorophore when controlling the plasmon resonance spectrum of nanoparticles can lead to corresponding optimal settings for obtaining maximum MEF values in the spectral region where the fluorophore does not emit or emits with minimal intensity [30].

In a recent study [31], the spectral dependence of the surface plasmon resonance of nanoparticles of various shapes (spheres, rods, disks, and crescent-shaped disks), representing various types of nanoantennas, was numerically studied over a wide spectral range of wavelengths from the red (650 nm) to the near-IR region in order to find the maximum MEF value. When searching for the maximum MEF for different orientations of the fluorescent molecule’s dipole moment, it was assumed that emission occurs at a single wavelength in the visible or near-IR region of the spectrum, which leads to unrealistic settings and MEF values.

In the proposed spectral approach, the controlling parameter is not only the spectral position of the plasmon resonance maximum and its spectral shape but also the spectral susceptibility of the fluorophore.

To optimize fluorescence enhancement, it is proposed to control the spectral properties of the plasmonic nanoparticle by varying the aspect ratio to overlap the absorption and fluorescence spectra of the dye molecule to achieve the maximum fluorescence enhancement factor. This spectral approach should address three main fluorescence enhancement processes: local field enhancement due to absorption by the fluorophore molecule and two competing processes caused by fluorescence enhancement due to an increase in the local optical field density, a decrease in the controlled gap between the nanoparticle and the fluorescent molecule, and the sharp quenching of the fluorescent molecule’s emission by the plasmonic nanoparticle (via the Forster mechanism) at distances of a few nanometers.

In this paper, we develop a traditional approach whereby the MEF optimization considers the properties of the fluorophore molecule at only two discrete wavelengths—the excitation maximum λ_ex_ and the emission maximum λ_em_ of the molecule. The proposed model provides a more realistic description of the processes of molecule irradiation and the generation of the fluorescence spectral response by introducing a new concept of effective properties of such parameters as field intensity enhancement factor ξ_eff_, quantum yield Y_eff_, and fluorescence enhancement factor K_flu eff_. The influence of the spectral properties of both the nanostructure plasmon and the fluorophore molecule on the optimal tuning of fluorescent complexes is investigated. Particular attention is paid to determining the spectral parameters of plasmon resonance—the absorption cross-sections C_abs_, scattering cross-sections C_sca_, and extinction cross-sections C_ext_—which can be found experimentally, as well as to calculating the intensity enhancement ξ of the excitation field in the near field of the plasmonic nanostructure relative to the extinction spectrum, which is “redshifted” compared to the other mentioned parameters. Numerical results of MEF optimization of fluorescent complexes are presented using TagRFP molecules in the vicinity of gold and silver nanorods as examples. It is demonstrated that recommendations for selecting nanorod dimensional parameters for tuning plasmon resonance, obtained based on the traditional and proposed models, can differ significantly.

## 2. Main Parameters of Localized Plasmon Resonance of Nanoparticles

The physical phenomenon of localized surface plasmon resonance (LSPR) occurs as a result of the interaction of photons of external optical radiation with the surface of metal nanoparticles, leading to the emergence of synchronized collective oscillations of valence electrons in the conduction band of metals. The most significant property of LSPR, which has found wide application in biomedicine, biochemistry, and numerous technical applications, is the sharp increase in the absorption of radiation by the nanoparticle in a certain wavelength range (known as resonant absorption). To describe this property of nanoparticles, an integral parameter, the absorption cross-section C_abs_, is often used [32].

Another parameter characterizing LSPR in the context of the efficiency of interaction of the irradiating radiation with the nanoparticle is the scattering cross-section C_sca_. In plasmon resonance, the scattering cross-section C_sca_, like C_abs_, increases sharply in a certain wavelength range [32].

The third important parameter of LSPR is the magnitude of the electric field intensity enhancement coefficient ξ = |E|^2^/|E_0_|^2^ in the vicinity of the nanoparticle (in the near-field zone), which plays a key role in the initialization and existence of many physical and physicochemical processes, including the phenomenon of fluorescence.

A common feature of the spectral dependences of each of these parameters is the existence of a local maximum associated with the LSPR phenomenon. However, depending on the properties of the nanoparticle material, as well as their shape and size, the position of each of these maxima on the wavelength axis can vary significantly. The physical mechanism for the observed “redshift” of the field enhancement maximum ξ and its possible magnitude, as applied to nanospheres, have been discussed in detail previously in [33,34,35].

To be able to compare theoretical and experimental data and establish their one-to-one correspondence, the extinction cross-section C_ext_ is also of practical interest; the measurement technique for this is widely known and well-developed [33].

**Figure 1:**
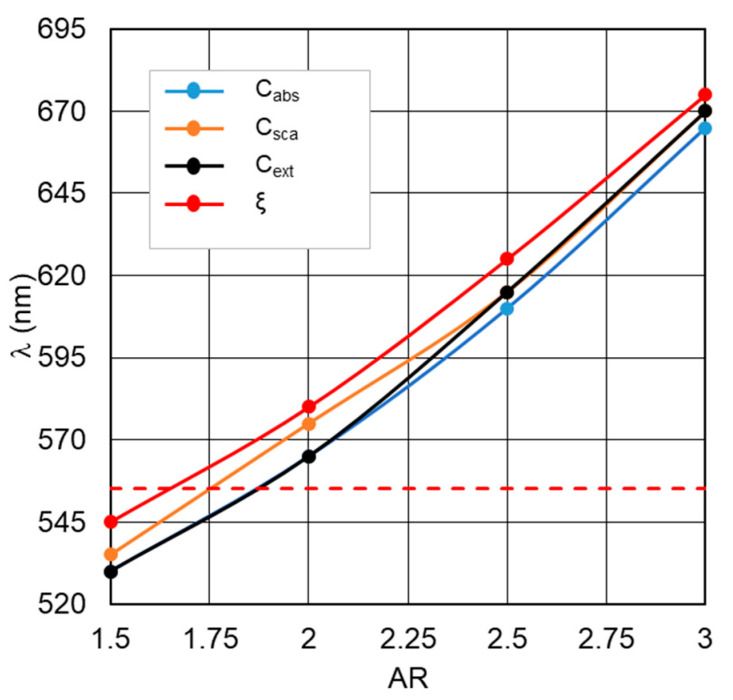
Dependence of the position of the maxima of C_abs_, C_sca_, C_ext_, and ξ gold nanorods (*d* = 50 nm) on the AR; the dotted line indicates the wavelength of maximum photosensitivity of the TagRFP fluorophore λ = 555 nm.

If the tuning capabilities of the plasmon resonance of nanospheres are limited to a relatively small range of the visible part of the optical spectrum of radiation, regardless of the measurement range of the nanosphere diameter, varying the dimensional parameters of the nanorods makes it possible to significantly expand the tuning range of the plasmon resonance from UV to IR [36]. Such dimensional control parameters of the studied nanorods are the cross-sectional diameter of the cylindrical middle part *d* and the form factor—aspect ratio (AR), equal to the ratio of the nanorod length *L*, including the hemispherical rounding of the end parts, to *d*. When choosing nanorods as the basic objects of study, the potential for tuning the position and absolute values of the plasmon resonance parameters by varying the AR is demonstrated for gold nanorods with *d* = 50 nm in Figure 1. In the case of silver nanorods, the magnitude of the noted “red” shift is noticeably lower.

The obtained results allow us to establish fundamentally important relationships between the positions of the spectral curve maximums for each of the four plasmon resonance parameters relative to one another when varying the dimensional parameters of the nanorods. This information plays a key role in the precision LSPR tuning procedure required for the actual MEF optimization procedure, since only one of the four main LSPR parameters is available for experimental verification. Without loss of generality, here and below, fluorescence issues are considered using the example of optimizing «plasmonic nanorod–TagRFP molecule» complexes [34].

The dependences of the LSPR parameter maxima on the gold nanorod size factors shown in Figure 1 indicate a nearly linear change in the wavelength maxima position with increasing nanorod length. Note that the maximum position of the electric field intensity enhancement parameter is redshifted relative to any other LSPR parameter. These patterns of one-to-one correspondence between the nanorod size factors and the maxima of various parameters enable precise tuning of plasmonic nanorod–TagRFP molecule complexes.

## 3. Traditional Monochromatic Model of MEF

In general, the optimization procedure for fluorescent «plasmonic nanorod–TagRFP molecule» complexes boils down to a comparative analysis of the interaction scenarios between the complex components while varying the nanoscale gap Δ between them. However, in our opinion, the traditional approach to modeling each of these scenarios is not without its drawbacks.

A characteristic feature of fluorescence is that even under monochromatic fluorophore irradiation, a fluorescence response (fluorescence signal) is observed over a relatively broad wavelength spectrum. When modeling MEFs to optimize the gain regime, a large number of parameters must be considered: the intensities and wavelengths of the excitation and emission signals, the shape, size, and material of the plasmonic nanoparticle, as well as the gap between it and the adjacent fluorescent molecule. Therefore, in general, optimization of the amplified characteristics (such as local field enhancement, quantum yield, and fluorescence signal gain) by specifically searching for the maxima of the corresponding distributions is difficult. For this reason, in most studies, the authors limited themselves to searching for the maxima of these distributions while considering only two fixed wavelengths of the excitation and emission signals. Our approach aims to eliminate this limitation.

**Figure 2:**
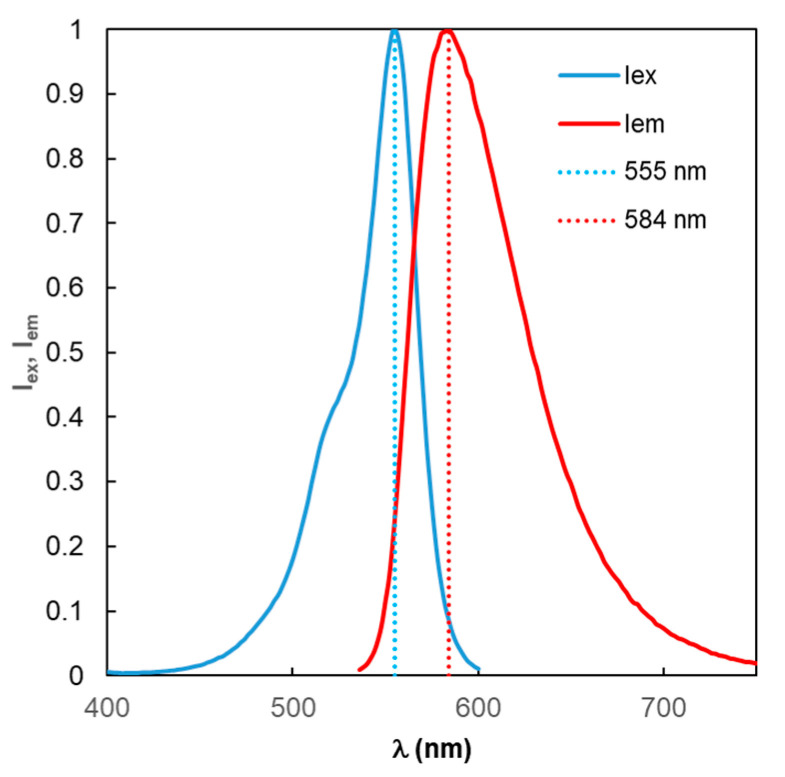
Excitation (*I*_ex_) and emission (*I*_em_) spectra of the TagRFP fluorophore. From here on, the vertical dotted lines labeled 555 nm and 584 nm indicate the wavelengths of the *I*_ex_ and *I*_em_ peaks, respectively.

It should be noted that at this stage of the study, the properties of the fluorophore molecules themselves are a mandatory element. As a rule, they have a complex structure [37,38]. In the fluorescence mode, the analysis of two spectra is relevant—the excitation spectrum *I*_ex_(λ) and the emission spectrum *I*_em_(λ), the maxima of which have a Stokes shift relative to each other. *I*_ex_(λ) and *I*_em_(λ) are normalized to their maximum values. A typical form of the spectra is shown in Figure 2 for the TagRFP fluorophore [39]. The wavelengths of the maxima of these two spectral curves are indicated by dotted vertical lines; these values are used as the main parameters in the generally accepted traditional model (TM) of fluorescence optimization. This approach can be conventionally designated as “monochromatic excitation, monochrome fluorescence at fixed wavelengths.” The properties of the fluorophore are also characterized by the value of the intrinsic quantum yield Y_0_, which rarely appears in traditional MEF modeling.

A certain basis for the widespread use of the TM approach can apparently be considered the fact that the greatest fluorescence emission power is concentrated in a fairly narrow wavelength range—in the vicinity of the maximum of the *I*_eM_(λ) function of the fluorophore. Indeed, for the fluorophore under consideration, TagRFP, as follows from an analysis of the *I*_em_(λ) curve in Figure 2, 35% of the power is concentrated in a wavelength range of 26 nm, where *I*_em_(λ) > 0.9. However, the remaining 65% of the fluorescence emission power is distributed in a wavelength range distant from λ = 584 nm. And this significant portion of the emission cannot be taken into account within the TM framework.

The numerical studies conducted in this paper demonstrate that the accepted “postulation” of achieving the highest possible values of both the electric field intensity enhancement and the quantum yield is not necessarily rigidly tied to these fixed wavelengths. Moreover, a shift relative to these wavelengths is typically observed, and only occasionally is the a priori condition met. The reason for this phenomenon lies precisely in the influence of the fluorophore’s spectral properties on the competition between three spectrally dependent processes: photoexcitation of the fluorophore molecules, emission of spectrally distributed radiation from the fluorophore molecules, and resonant quenching of this radiation at extremely small distances between the plasmonic nanoparticle and the fluorophore molecule.

To clarify the differences between the TM and the proposed refined model (RM) for MEF optimization, we will now outline the main assumptions, as well as the target values in both cases, first for TM, and then for RM.

TM:

(1) Monochromatic excitation at a fixed wavelength λ_ex_, corresponding to the maximum of the experimental curve *I*_ex_.
(2) Emission analysis at a fixed wavelength λ_em_, corresponding to the maximum of the experimental curve *I*_em_.
(3) Optimality criterion—the maximum fluorescence enhancement coefficient *K*_flu_ under constraints (1) and (2) specified above.

## 4. Development of a Model for Simulating the Spectral Fluorescence Response

The proposed RM includes:

(1) Monochromatic excitation at a wavelength λ, determined based on the condition of achieving a maximum of the modified electric field intensity function ξ_eff_ = |**E**|^2^/|**E**_0_|^2^, determined taking into account the spectral excitation function of the fluorophore *I*_ex_, in the range of variation «excitation wavelength—the dimensional parameters of the plasmonic nanoparticle». We will call ξ_eff_ the effective field intensity enhancement factor.
(2) Determination of the effective emission parameters (effective radiation losses Γ_rad eff_, effective nonradiation losses Γ_nr eff_, and effective quantum yield *Y*_eff_) in the fluorescence wavelength range, taking into account the spectral function *I*_em_.
(3) The optimality criterion is the maximum fluorescence signal enhancement (effective fluorescence enhancement factor *K*_flu eff_) in the range of variation «emission wavelength—the dimensional parameters of the plasmonic nanoparticle».

Our modeling was based on the solution of the wave equation written for the electric field in the form [40]

(1) $$\mathrm{rot}\mu^{-1}\mathrm{rot}E-\omega^{2}\varepsilon E=-i\omega j_{0}.$$

The solution of Equation (1) was carried out numerically using the COMSOL Multiphysics^®^ (https://cn.comsol.com/) computing package (Wave Optics module) [41]. As a result, the spatial distributions of the complex vectors of the electric field strength **E** and magnetic field strength

(2) $$H=i{\left(\omega \mu \right)}^{-1}\mathrm{rot}E,$$

corresponding to the monochromatic excitation mode at frequency ω of a homogeneous, isotropic scatterer with permittivity functions ε and magnetic permeability μ; the value **j**_0_ denotes the given (external) conductivity current density of the object.

Important energy characteristics are the power of scattered energy

(3) $$W_{\mathrm{rad}}=\iint_{A}SdA,$$

and absorbed energy

(4) $$W_{\mathrm{nr}}=\underset{V}{∭}Qdv.$$

In (3), the Poynting vector (averaged over the period of optical oscillations) **S** = 0.5ReE* × **H** is integrated over the sphere of area *A* enclosing the object, and in (4), the specific power of radiation absorption

*Q* = 0.5[ωε_0_ε″|**E**|^2^ + Re(E*⋅**j**_0_)], (5)

is integrated over the volume *V* of the object. In (5), the quantity **j**_0_ denotes the given (external) density of the conductivity current of the object, simulating the source of the fluorescent signal, and ε″ is the imaginary part of its permittivity.

To analyze the fluorescence mode, we considered two methods of field excitation:

(1). At the stage of excitation of the object by an irradiating field of intensity *I*_0_ with a given vector of complex amplitude **E**_0_ at a frequency ω_ex_, the homogeneous equation (1) was solved (this is an equation with a zero right-hand side, i.e., at **j**_0_ = 0) and the distributions of local values of the vectors **E**, **H** and the corresponding energy characteristics (3)–(5) were calculated. If, within the scatterer, the irradiating field **E**_0_ can be locally approximated by a plane wave, then scattering and absorption are usually characterized by the corresponding cross-sections [21]. Namely, the scattering cross-section

(6) $$C_{\mathrm{sca}}=W_{\mathrm{rad}}/I_{0},$$

absorption cross-section

(7) $$C_{\mathrm{abs}}=W_{\mathrm{nr}}/I_{0},$$

and the extinction cross-section *C*_ext_. Note that in the experiment, *C*_ext_, *C*_sca_ are found, and for a non-absorbing environment, the cross-section *C*_abs_ is calculated as [42]

*C*_abs_ = *C*_ext_ − *C*_sca_. (8)

The method we propose to take into account the spectral dependence of excitation consists of replacing the formula for the local field gain factor

(9) $$\xi =|E{|}^{2}/|E_{0}{|}^{2},$$

the value ξ with ξ_eff_:

ξ_eff_ = *I*_ex_⋅ξ. (10)

(2). At the stage of emission of the fluorescent signal at the frequency ω_em_, the inhomogeneous Equation (1) was solved, and the distributions of local values of the vectors **E**, **H** and the corresponding energy characteristics were calculated directly using Formulas (3)–(5). In contrast to the excitation stage, the fields **E**, **H** were induced not by the irradiating field with a given complex amplitude **E**_0_, but by a given (external) density of the conductivity current of the object **j**_0_. This is equivalent to setting the corresponding external dipole moment p_0_ [41], simulating the source of the fluorescent signal, which has a spontaneous nature and, as in works [9,21,25,42], is considered by us as a point object.

An important characteristic of the fluorescence regime is the quantum yield *Y* [10,21,25]. To calculate this value, we proposed a modified formula that takes into account the spectral dependences of the fluorescent molecule’s response:

(11) $$Y_{\mathrm{eff}}=\frac{\Gamma_{\mathrm{rad}\mathrm{eff}}}{\Gamma_{\mathrm{rad}\;\mathrm{eff}}+\Gamma_{\mathrm{nr}\;\mathrm{eff}}+1/Y_{0}-1},$$

Here

(12) $$\Gamma_{\mathrm{rad}\;\mathrm{eff}}=I_{em}W_{\mathrm{rad}}/(\hbar \omega_{\mathrm{em}}),$$

and

(13) $$\Gamma_{\mathrm{nr}\;\mathrm{eff}}=I_{\mathrm{em}}W_{\mathrm{nr}}/(\hbar \omega_{\mathrm{em}}),$$

respectively, the effective rates of radiative and non-radiative relaxation of the “nanoparticle—fluorophore molecule” complex, normalized to $\Gamma_{\mathrm{rad}}^{0}=W_{\mathrm{rad}}^{0}/(\hbar \omega_{\mathrm{em}})$—the rate of radiative relaxation of an isolated fluorophore molecule with the value of its own quantum yield *Y*_0_.

Then, the resulting formula for the fluorescence enhancement coefficient [10,21,25] is also transformed and can be written as

*K*_flu eff_ = ξ_eff_ ⋅ *Y*_eff_. (14)

## 5. Numerical Simulation Results and Discussion

In order to demonstrate the comparative effectiveness of the traditional TM and proposed RM approaches to optimizing MEF, we will explain the sequence of computational procedures when calculating the desired parameters in the two cases indicated. The fluorescent complex “nanorod—TagRFP molecule” was chosen as a model object, and options for constructing fluorescent complexes based on gold and silver nanorods were considered; calculations were carried out with water parameters as the environment.

The traditional approach involves performing three consecutive steps:

- Determination by Formula (9) of the dependence of the amplification coefficient of the intensity of the exciting field ξ = |E|^2^/|E_0_|^2^ on the length λ_ex_ when varying the dimensional parameters of the nanostructure (in our case, the AR is nanorod form factor) at a given gap Δ;
- Determination of the dependence of the quantum yield Y on the emission of a dipole of a fluorophore molecule at a length of λ with varying dimensional parameters of the nanostructure and a given gap Δ, taking into account the intrinsic quantum yield of the molecule Y_0_.
- Based on the found dependencies ξ and Y, the value of the dimensional parameter AR is determined, at which the maximum fluorescence gain coefficient *K*_flu_ is achieved.

**Figure 3:**
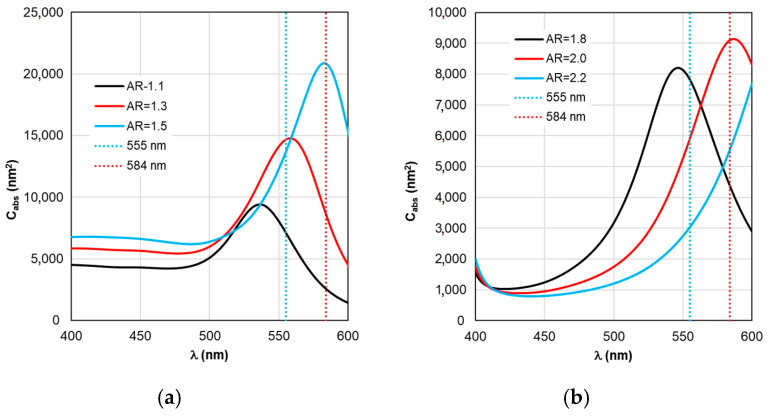
Dependence of the absorption cross-section of C_abs_ on the irradiation wavelength with varying AR form factor. Here and in the figures below, (**a**) corresponds to a fluorescent complex based on gold nanorods; (**b**) corresponds to a complex based on silver nanorods.

Figure 3 shows the results of calculating the absorption cross-section *C*_abs_ of plasmonic nanorods of the fluorescent complex when the AR form factor is changed. It can be seen that with an increase in AR by 0.2 units, the peak of plasmon resonance of gold nanorods shifts by 25 nm (see Figure 3a), and silver nanorods—by 40 nm (see Figure 3b). The wavelength range of the abscissa axis in Figure 3 corresponds to the photosensitivity range of TagRFP.

**Figure 4:**
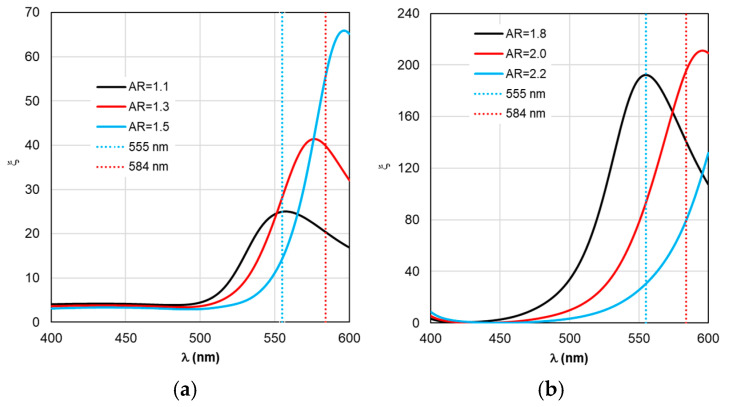
Spectral distribution of the field intensity enhancement factor ξ, calculated within the TM framework, when changing the AR form factor.

**Figure 5:**
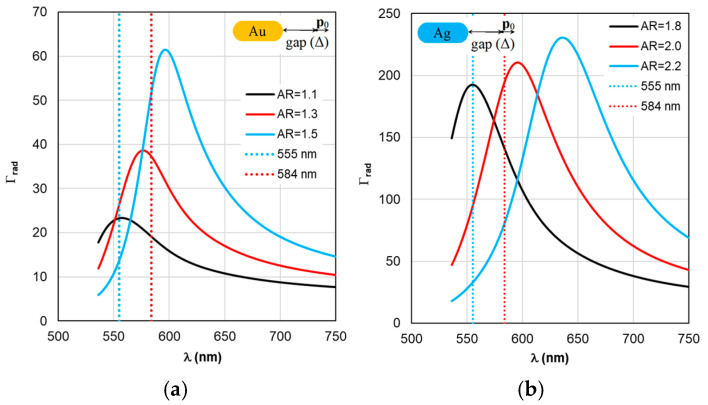
Spectral distribution of the radiative relaxation rate Γ_rad_, calculated within the TM framework, with a change in the AR form factor.

**Figure 6:**
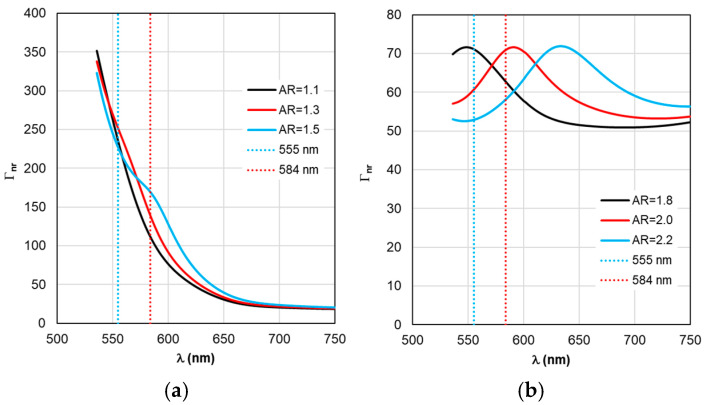
Spectral distribution of the non-radiative relaxation rate Γ_nr_, calculated within the TM framework, with a change in the AR form factor.

**Figure 7:**
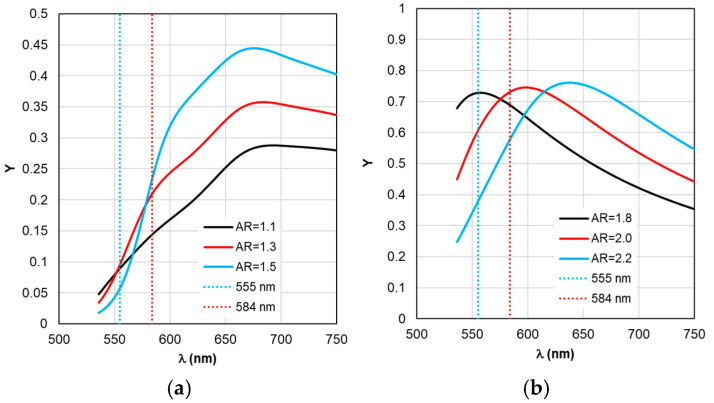
Spectral distribution of the quantum yield Y, calculated within TM with changing form factor AR.

**Figure 8:**
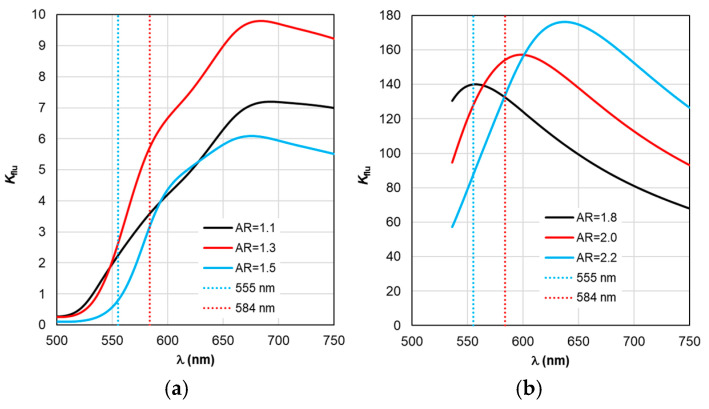
Spectral distribution of the fluorescence enhancement coefficient *K*_flu_, calculated within the TM framework, with a change in the AR form factor.

The sequence of calculations of the defining parameters of fluorescence by TM is illustrated by the calculation results shown in Figure 4, Figure 5, Figure 6, Figure 7 and Figure 8. The calculations were performed with an optimal “nanorod—TagRFP molecule” gap of Δ = 6 nm in the case of a fluorescent complex based on gold nanorods and Δ = 3 nm in the case of a fluorescent complex based on silver nanorods. The results of TM calculations of the field enhancement coefficient ξ, quantum yield Y, and fluorescence enhancement coefficient K_flu_ as functions of the gap Δ are shown in Figure S1 for nanocomplexes based on gold nanorods. Similar dependences of the parameters on the gap Δ for nanocomplexes based on silver nanorods are shown in Figure S2. These data allow us to estimate the sensitivity of the K_flu_ to the gap parameter Δ. The differences in the MEF TagRFP results found for TM and RM when varying the AR of gold nanorods can be noted from a comparative analysis of the distributions on Figures S3 and S4. They mainly relate to changing the nature of the dependencies Y and Y_eff_, K_flu_ and K_flu eff_. The configuration of the nanocomplex with the dipole orientation perpendicular to the spherical surface of the nanorod and excitation of the longitudinal LSPR is effective, so it was chosen for the study in this work (the diagram is shown in Figure 5a,b).

Despite the fact that the distributions of calculated values in Figure 4, Figure 5, Figure 6, Figure 7 and Figure 8 are given in the full range of excitation/emission wavelengths of the fluorophore, we take into account the basic position of the TM concept—the calculations result in discrete values of the field intensity gain ξ at λ_ex_ = 555 nm (see Figure 4), the rate of radiative Γ_rad_ and nonradiative Γ_nr_ relaxation of the complex (see Figure 5 and Figure 6), the quantum yield Y (see Figure 7), and the fluorescence gain *K*_flu_ (see Figure 8) at λ_em_ = 584 nm.

A comparative analysis of the curves in Figure 3 and Figure 4 reveals a certain correlation between the position of the Cabs and ξ peaks: at the same AR values, the peaks of the ξ curves shift along the wavelength to the “red” side by about 20 nm in the case of gold nanorods and 8 nm in the case of silver nanorods compared to the position of the corresponding C_abs_ peaks. At the same time, the distance between the neighboring peaks of C_abs_ and the neighboring peaks of ξ remains the same. The same trends in the position of peaks synchronized with the C_abs_ peaks are characteristic of the Γ_rad_ in Figure 5 in the case of gold and silver nanorods.

The situation changes somewhat when considering the spectral dependences of Γ_nr_. While for complexes based on silver nanorods, the above-mentioned pattern of synchronous changes in the position of the Γ_nr_ and C_abs_ peaks remains the same (see Figure 3b and Figure 6b), for complexes based on silver nanorods, Γ_nr_ peaks in the fluorescence range are not observed (see Figure 6b). Γ_nr_ increases monotonously as it moves towards short wavelengths, and there is no correlation with the C_abs_ resonance. Note that the high level of Γ_nr_ in Figure 6 is the same as Γ_rad_ (see Figure 5) at the edges of the fluorescence range (this is a consequence of not taking into account the spectral properties of the fluorophore shown in Figure 2), which is the main disadvantage of TM, leading to a contradiction in the physics of the spectrally selective fluorescence process. The spectral dependences Γ_rad_ and Γ_nr_ found lead to the determination of the corresponding curves for the quantum yield Y shown in Figure 7. Using the values ξ at λ_ex_ = 555 nm from the results in Figure 4 and the dependence Y from Figure 4, it is possible to determine the gain coefficient *K*_flu_ from the curves plotted in Figure 8 at λ_em_ = 584 nm. Thus, as a result of modeling using TM for complexes based on gold nanorods, as follows from Figure 8a, the choice of a nanorod with AR = 1.3, which provides *K*_flu_ = 5.6 when excited at a wavelength of λ_ex_ = 555 nm, is recommended as the optimal configuration. When using silver nanorods, the optimal configuration is recommended to choose a nanorod with AR = 2.0, which provides *K*_flu_ = 152 when excited at a wavelength of λ_ex_ = 555 nm.

**Figure 9:**
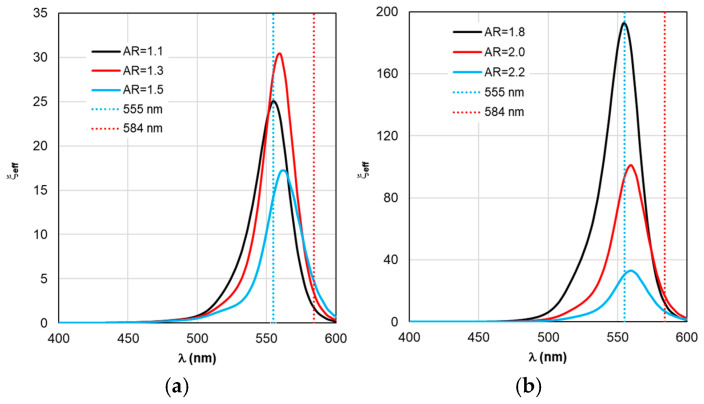
Spectral distribution of the effective field intensity enhancement factor ξ_eff_, calculated within the RM framework, with a change in the AR form factor.

**Figure 10:**
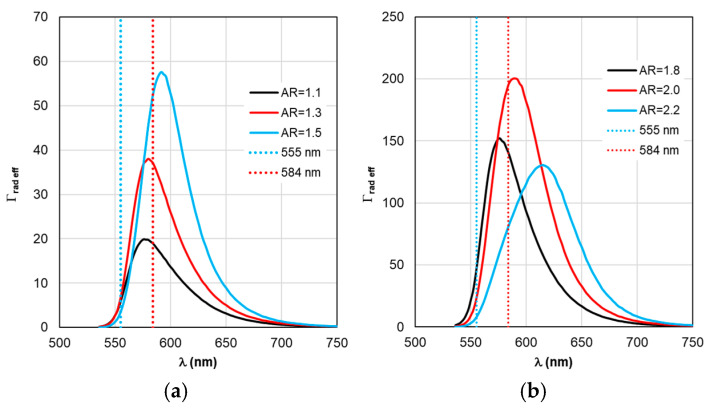
Spectral distribution of the effective radiative relaxation rate Γ_rad eff_, calculated within the RM framework, with a change in the AR form factor.

**Figure 11:**
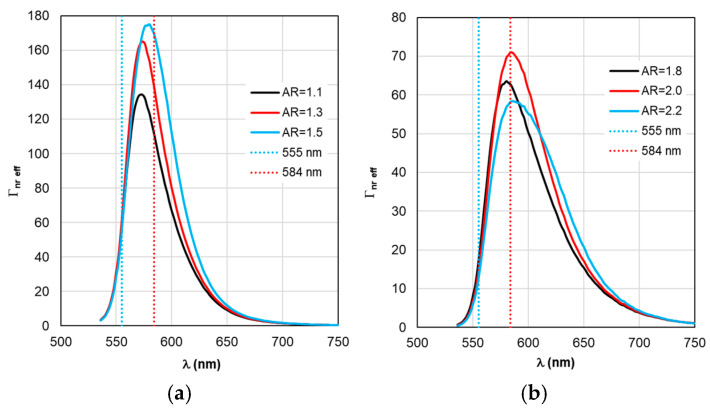
Spectral distribution of the effective non-radiative relaxation rate Γ_nr eff_, calculated within the RM framework, with a change in the AR form factor.

**Figure 12:**
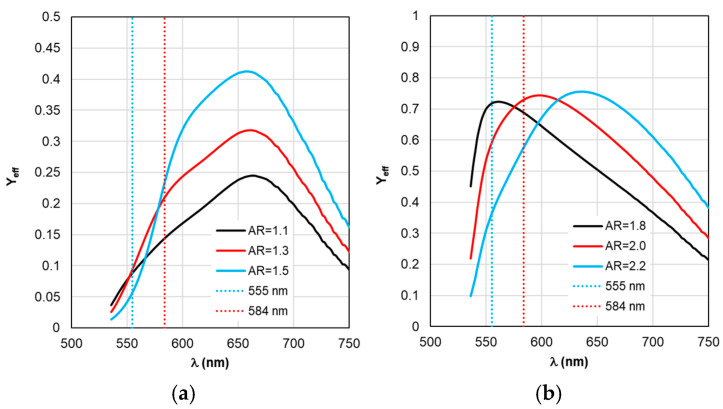
Spectral distribution of the effective quantum yield Y_eff_, calculated within RM with changing form factor AR.

**Figure 13:**
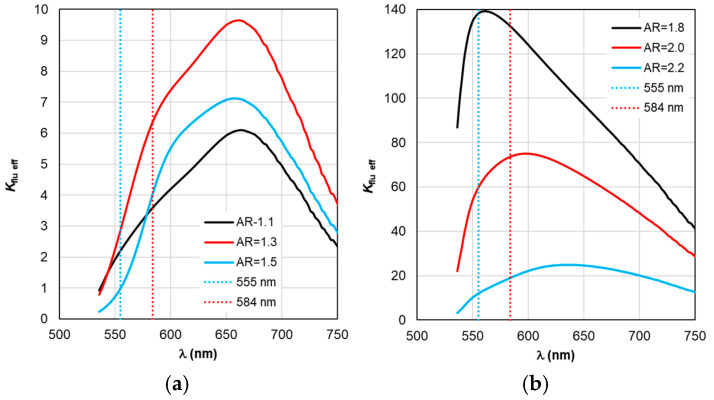
Spectral distribution of the effective fluorescence enhancement coefficient *K*_flu eff_, calculated within the RM framework, with a change in the AR form factor.

Fluorescence optimization according to the proposed approach using RM MEF involves performing calculations in the same sequence. However, fundamental differences arise from the introduction of new concepts: effective field enhancement factor ξ_eff_, effective quantum yield Y_eff_, and effective fluorescence enhancement factor *K*_flu eff_. To determine the effective excitation fields and fluorescence emission functions, Formulas (9–14) are used, which include the fluorophore spectral property functions *I*_ex_ and *I*_em_ (shown in Figure 3). The results of calculations using RM MEF are presented in Figure 9, Figure 10, Figure 11, Figure 12 and Figure 13.

The main differences between the calculation results for ξ_eff_, Γ_rad eff_, and Γ_nr eff_ in Figure 9, Figure 10 and Figure 11 are:

(i) spectral localization (the curves describing the dependences of the physical process parameters are defined strictly in the excitation/emission range of the fluorescent molecule);
(ii) a correlation between the shifts in the ξ_eff_, Γ_rad eff_, and Γ_nr eff_ peaks and the shifts in the C_abs_ peaks (see Figure 3) exists, but is expressed to a much lesser degree, while the differences in the actual values of the ξ_eff_, Γ_rad eff_, and Γ_nr eff_ maxima are significant;
(iii) the physical interpretation of the obtained results corresponds to the description of the spectral sensitivity upon excitation and the spectral distribution of fluorescent radiation upon emission of fluorophore molecules.

Comparing ξ_eff_ in Figure 9a and C_abs_ in Figure 3a reveals that for gold nanorod-based complexes, the trend for ξ_eff_ peaks to move synchronously with C_abs_ peaks remains. However, the 25 nm distance between C_abs_ peaks decreases by an order of magnitude relative to adjacent ξ_eff_ peaks. For silver nanorod-based complexes, the distance between ξ_eff_ peaks decreases by a factor of 40. This nontrivial result can be termed the ξ_eff_ stabilization effect.

Of practical interest is the fact that there appears to be no universal recommendation for tuning the LSPR when selecting the nanorod size parameter. Recommendations may vary depending on the optical properties of the nanorod material. For example, a comparison of Figure 3a and Figure 9a shows that maximum excitation of the TagRFP molecule (maximum ξ_eff_) in complex with a gold nanorod is achieved by tuning the C_abs_ peak to a wavelength of 559 nm, which exceeds λ_ex_ = 555 nm. In the case of a complex based on silver nanorods (see Figure 3b and Figure 9b), to ensure maximum excitation, it is necessary to adjust the C_abs_ wavelength in the negative direction—to a wavelength of 542 nm. The RM calculation results in each case considered make it possible to determine the direction and magnitude of the LSPR plasmon resonance detuning depending on the material properties and the size of the nanorods.

As for Γ_rad eff_ and Γ_nr eff_ in Figure 10 and Figure 11, the distance between adjacent peaks of Γ_rad eff_ of complexes with gold nanorods is 11 nm, with silver nanorods −15 nm, and between peaks of Γ_nr eff_ −5 and 2 nm, respectively.

Based on the calculation results of Y_eff_ in Figure 12 and ξ_eff_ in Figure 9, the *K*_flu eff_ dependencies presented in Figure 13 are obtained. The dependence analysis in Figure 13a shows that when using RM, it is recommended to choose AR = 1.3 as the optimal configuration of the complex based on gold nanorods, which has a maximum *K*_flu eff_ = 9.7 (*K*_flu eff_ = 6.4 at a wavelength of λ_ex_ = 584 nm) when excited at a wavelength of λ_ex_ = 555 nm. This result is higher compared to the one obtained by using TM. It should be noted that the results of calculating *K*_flu eff_ for other values of the AR form factor differ from those obtained using TM (see Figure 8a) not only quantitatively but also qualitatively.

It follows from the data in Figure 13b that for complexes based on silver nanorods, the optimal configuration is recommended to choose a nanorod with AR = 1.8 (differs from the recommendation according to the TM results), which provides a maximum *K*_flu eff_ = 140 when excited at a wavelength of λ_ex_ = 555 nm. Using the recommended size of nanorods according to TM AR = 2.0 leads to a twofold decrease in the maximum value of *K*_flu eff_.

**Figure 14:**
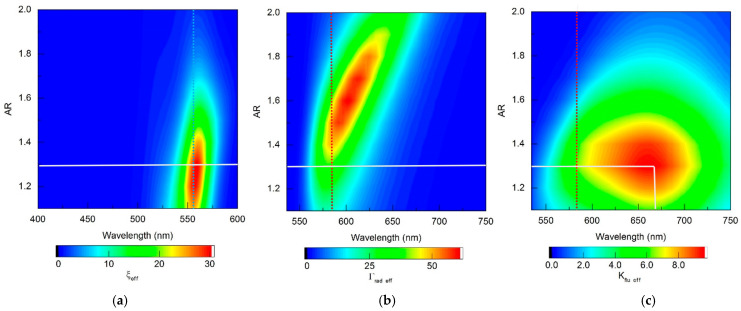
Distribution maps of (**a**) the effective field intensity enhancement factor ξ_eff_, (**b**) the effective radiative relaxation rate Γ_rad eff_, and (**c**) the effective fluorescence enhancement coefficient *K*_flu eff_, calculated within the RM framework for complexes based on gold nanorods.

**Figure 15:**
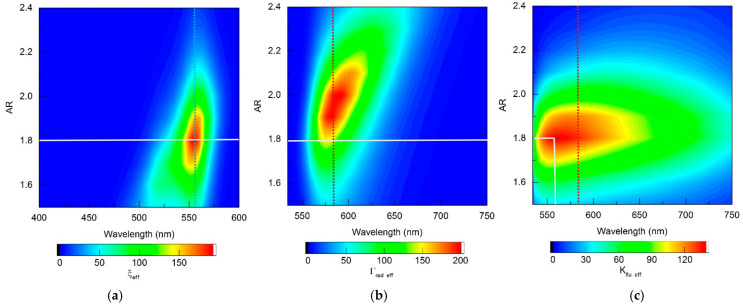
Distribution maps of (**a**) the effective field intensity enhancement factor ξ_eff_, (**b**) the effective radiative relaxation rate Γ_rad eff_, and (**c**) the effective fluorescence enhancement coefficient *K*_flu eff_, calculated within the RM framework for complexes based on silver nanorods.

The refinements obtained using RM for choosing the optimal configuration of fluorescent complexes seem significant. The proposed technique provides localization and, ultimately, unambiguous determination of the zone of optimal parameters of fluorescent complexes, taking into account the spectral sensitivity of the fluorophores used, which is illustrated by distribution maps in Figure 14 (gold nanorod-based complexes) and Figure 15 (silver nanorod-based complexes). As can be seen from Figure 14 and Figure 15, all distributions of the sought quantities obtained within the framework of the RM model are localized in the “wavelength—AR” coordinates. The zones of high values of ξ_eff_, Γ_rad eff_, and K_flu eff_ are limited to ellipsoid-shaped areas, the maximum position in each of them is easily determined unambiguously. The adopted procedure for selecting the optimal AR parameter of the fluorescent complex is to find the maximum K_flu eff_, as shown in Figure 14c and Figure 15c (the maximum is indicated by the intersection point of vertical and horizontal solid white lines). The choice of AR determines the spectral distribution of the fluorescent radiation (defined by the distribution of the function Γ_rad eff_ on the horizontal solid white line in Figure 14b and Figure 15b). The optimal excitation wavelength is selected based on the achievement of the maximum value of the ξ_eff_ function on the horizontal solid white line in Figure 14a and Figure 15a. It should also be noted that the high ξ_eff_ values in Figure 14a and Figure 15a have an elliptical shape that is slightly inclined relative to the vertical. An obvious consequence of this is that, with varying AR, the position of the ξ_eff_ maximum may not coincide with λ_ex_ = 555 nm, indicated by the vertical blue dotted line. The same is true for the Γ_rad eff_ maximum relative to λ_ex_ = 584 nm, indicated by the vertical red dotted line in Figure 14b and Figure 15b.

Due to the fact that RM implements a more general approach, providing information on the spectral distribution of Γ_rad eff_ over the entire range of fluorophore emission (see Figure 14b and Figure 15b) in comparison with TM, it seems advisable to further build a strategy for optimizing fluorescent complexes using some integral estimates as a criterion. Thus, for example, for a selected AR, the integral of the normalized function Γ_rad eff_ over the range of fluorophore emission wavelengths, taking into account the spectral dependence of the photon energy, multiplied by the maximum ξ_eff_ for the same AR, can be taken as an estimate of the gain factor of the total power of the fluorescent signal.

The influence of spacer molecules and dielectric (including absorbing) shells on the plasmonic properties of metal nanoparticles is a pressing issue and has attracted the attention of many researchers. In particular, the results [34] of a numerical study of the local field enhancement in a MEF nanosphere with a two-layer sandwich shell (a layer of a biotin spacer and TagRFP fluorophore molecules) can be cited. It was shown that this type of coating leads to a redshift of the nanoparticle’s plasmon resonance in the near field and a decrease in ξ compared to the case of an uncoated plasmonic particle. These features are consistent with the findings of other authors [43,44]. Chemical interface damping (CID) is also a known effect associated with the absorption of molecules on the surface of a metal nanoparticle. Taking CID into account leads to a broadening of the plasmon band due to a decrease in the LSPR quality factor [45]. The question of conformation requires the use of experimental data and/or numerical molecular dynamics simulations. In [46], it was shown that a change in the spatial configuration of a fluorescent protein can indeed lead to a change and a significant shift in the excitation spectrum toward the red region.

The above-mentioned questions were not the focus of this study for one reason. The primary goal was to investigate how the plasmonic spectrum interacts with the spectral properties of the fluorophore itself. Clearly, the influence of the shell, linker molecules, and spatial configuration of the fluorophore molecule will alter this interaction, shifting both the plasmonic band and the maximum of the fluorophore’s excitation and emission spectrum. However, the methodological recommendations formulated in this study will remain relevant in this case.

An analysis of numerous experimental studies on MEF [45,47,48,49,50,51] shows that all authors agree on the effectiveness of using plasmonic nanoparticles to enhance the fluorescence response of fluorophores. However, there is no single universal algorithm for tuning LSPR to ensure optimal fluorescence. For example, the authors of [28] observed, on average, the brightest fluorescence from dyes attached to metal nanoparticles that have an LSPR scattering peak ~40–120 meV higher in energy than the emission peak of the fluorophore. These results, they believe, should prove useful for understanding and optimizing metal-enhanced fluorescence. However, they note that this recommendation is valid only for two of the three fluorophores studied. Apparently, it is quite natural to expect that, depending on the width, shape, and position of the fluorophore excitation/emission spectra, the “rules” for tuning nanocomplexes will be individual. We hope that the proposed RM will be useful in this regard.

In our opinion, the authors’ report [47] on the observed effect of the relationship between the LSPR peak position and the shift in the spectral composition and the position of the fluorescent signal peak is quite interesting. Qualitatively, the nature of the transformation of the fluorescent signal spectrum is well described by the results of our calculations using RM (see, for example, Figure 10a,b). Experimental studies [45,48] also confirm the previously identified pattern. The authors of [48,49] noted, as an effective configuration, a nanocomplex with the dipole orientation perpendicular to the spherical surface of the nanorod and the excitation of the longitudinal LSPR. This particular configuration was chosen for the study in this paper (the diagram is shown in Figure 5a,b). A quantitative comparison of the results of theoretical calculations and experimental studies requires special painstaking and time-consuming preparation of the work, which is planned for the future. As a good example, we can mention the approbation of model software in [50,51] by comparing numerical results with the solution of analytical problems.

A formalized methodology for MEF optimization was presented in [50,51], which is certainly an achievement. By choosing quantum efficiency as the objective function to be maximized under pre-selected Purcell factor criteria, the authors implemented optimization of plasmonic configurations based on nanorods and dimers. At the same time, the implemented concept is based on the use of a monochromatic fluorophore excitation/emission approach. The model for optimizing the MEF mode proposed in this paper, with detailed consideration of the shape of the fluorophore excitation and emission spectra, appears to expand the capabilities of the numerical description of MEF processes.

The universality of the proposed approach is due to the fact that the properties of an arbitrarily selected fluorophore are described by two spectral functions, *I*_ex_ and *I*_em_. Depending on the width, shape, and position of the peaks of these functions, the nature of the interaction in the nanocomplex can vary quite widely. Methods are known for tuning the transverse LSPR to the *I*_ex_ peak, and the longitudinal LSPR to the *I*_em_ peak. It appears that within the framework of the proposed RM, under certain conditions, it is possible to synthesize a complex, for example, with two fluorescence response peaks.

## 6. Conclusions

1. An RM is proposed that takes into account the spectral selectivity of the excitation/emission properties of a fluorophore in a finite wavelength range, which is universal with respect to arbitrary fluorophores used in the construction of fluorescent complexes.
2. The position of the maximum intensity of the LSPR field in the near zone, used for spectral tuning of the fluorescent complex, has a redshift relative to the C_abs_ peak: for the studied gold nanorods, it is 22 nm, and for silver nanorods, −8 nm. This information is important for precision tuning of the field maximum based on the results of experimental measurements of absorption parameters.
3. The spectral sensitivity of the fluorophore molecule leads to the effect of spectral stabilization ξ_eff_: the change in the position of the maximum of the ξ LSPR field intensity with a change in the nanorod form factor is several times greater than the change in the position of the maximum ξ_eff_.
4. The maxima of the spectral dependences of the field enhancement coefficient, radiative and non-radiative relaxation rates, found using RM, as a rule, do not coincide with the maxima of the functions *I*_ex_, *I*_em_, and the nature of the curves corresponds to the physical meaning of the modeled processes.
5. It is shown that recommendations for choosing optimal parameters of fluorescent nanocomplexes based on RM results can significantly (several times) improve the parameters of synthesized fluorescence nanocomplexes compared with ones obtained using the recommendations on TM. A positive factor of practical significance is information on the dependence of the fluorescence enhancement factor over the entire wavelength range of the fluorescence signal.

## Data Availability

The original contributions presented in this study are included in the article/Supplementary Materials. Further inquiries can be directed to the corresponding author.

## Supplementary Materials

The following supporting information can be downloaded at: https://www.mdpi.com/article/10.3390/ma19061258/s1, including Figure S1: Dependences of the field enhancement coefficient ξ, quantum yield Y (fragment on the left), fluorescence enhancement coefficient K_flu_ (fragment on the right) vs. gap Δ (designated as Distance); TM, “AuNR-TagRFP”; Figure S2: Dependences of the field enhancement coefficient ξ, quantum yield Y (fragment on the left), fluorescence enhancement coefficient K_flu_ (fragment on the right) vs. gap Δ (designated as Distance); TM, “AgNR-TagRFP”; Figure S3: Dependence of: (a) field enhancement coefficient ξ, (b) quantum yield Y, (c) fluorescence enhancement coefficient K_flu_ vs AR; TM, “AuNR-TagRFP”; Figure S4: Dependence of: (a) field enhancement coefficient ξ_eff_, (b) quantum yield Y_eff_, (c) fluorescence enhancement coefficient K_flu eff_ vs AR; RM, “AuNR-TagRFP”.

## Abbreviations

MEF	Metal-enhanced fluorescence
DNA	Deoxyribonucleic acid
LSPR	Localized surface plasmon resonance
AR	Aspect ratio
TM	Traditional model
RM	Refined model
