# Supplementary material for: Metal-Enhanced Fluorescence of Nanocomplexes
Source: Materials (Basel). 2026 Mar 22;19(6):1258. doi: 10.3390/ma19061258 (PMC13027601; doi:10.3390/ma19061258)
Supplement: Supplementary file 1 [file materials-19-01258-s001.zip › materials-4198826-supplementary.pdf]

Supporting Information

Metal-enhanced fluorescence of nanocomplexes

A.N. Yakunin, [1*] S.V. Zarkov, [1] Yu.A. Avetisyan, [1] G.G. Akchurin, [1,2] V.V. Tuchin [1,2,3]

[1] Laboratory of Laser Diagnostics of Technical and Living Systems, IPMC RAS, FRC "Saratov Scientific Centre of the RAS," Saratov, Russia; yuaavetisyan@mail.ru (Y.A.A.); akchuringg@mail.ru (G.G.A.); tuchinvv@mail.ru (V.V.T.)

[2] Institute of Physics and Science Medical Center, Saratov State University, Saratov, Russia

[3] Laboratory of Biophotonics, Tomsk State University, Tomsk, Russia, Russia

* Correspondence: anyakunin@mail.ru; Tel.: +7-845-222-2376

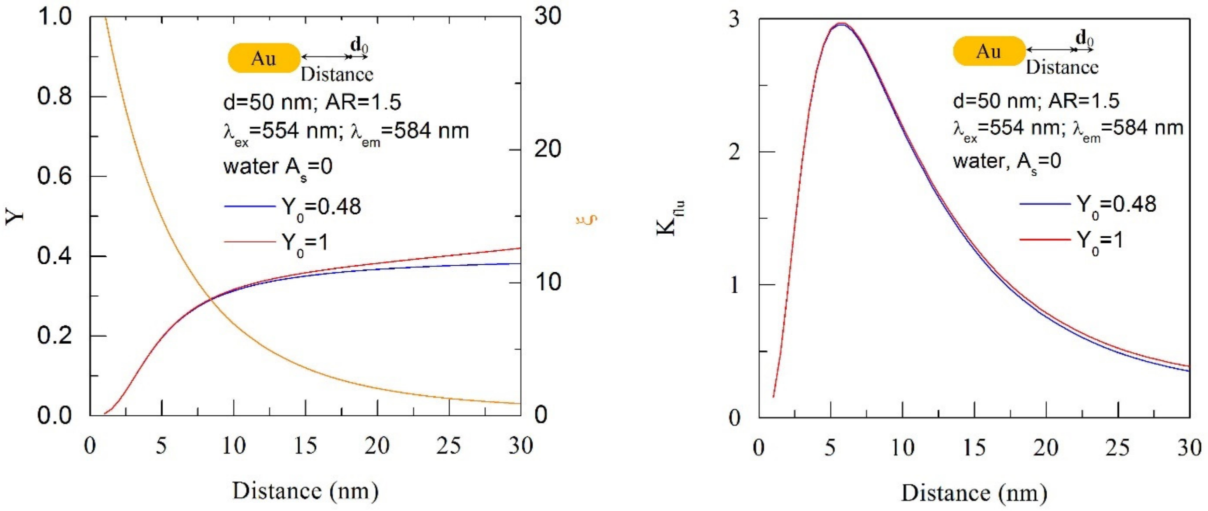

Figure S1. Dependences of the field enhancement coefficient ξ, quantum yield Y (fragment on the left), fluorescence enhancement coefficient K_{flu} (fragment on the right) vs. gap Δ (designated as Distance); TM, "AuNR - TagRFP".

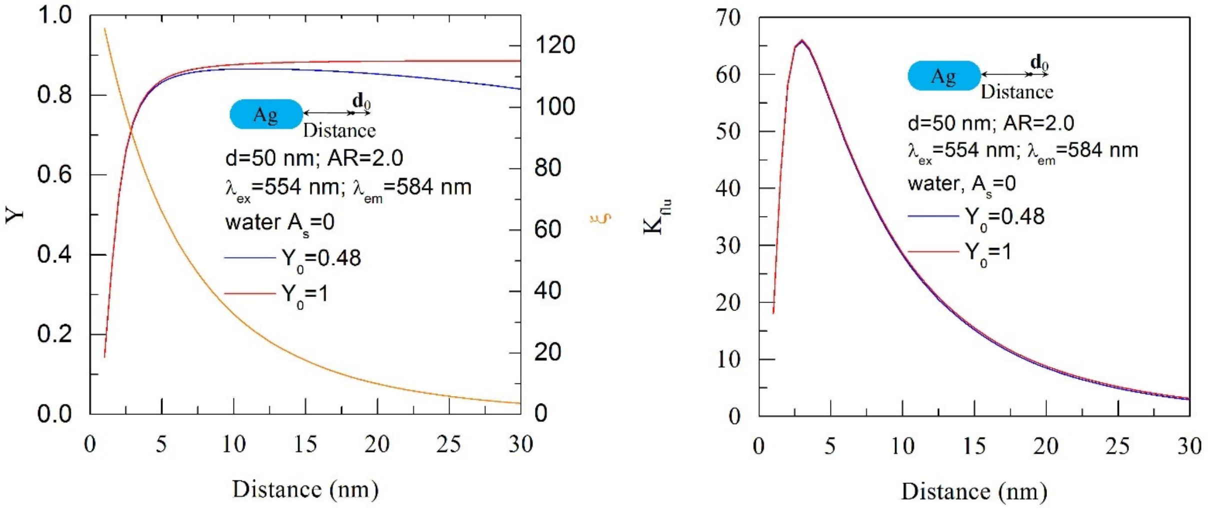

Figure S2. Dependences of the field enhancement coefficient ξ, quantum yield Y (fragment on the left), fluorescence enhancement coefficient K_{flu} (fragment on the right) vs. gap Δ (designated as Distance); TM, "AgNR - TagRFP".

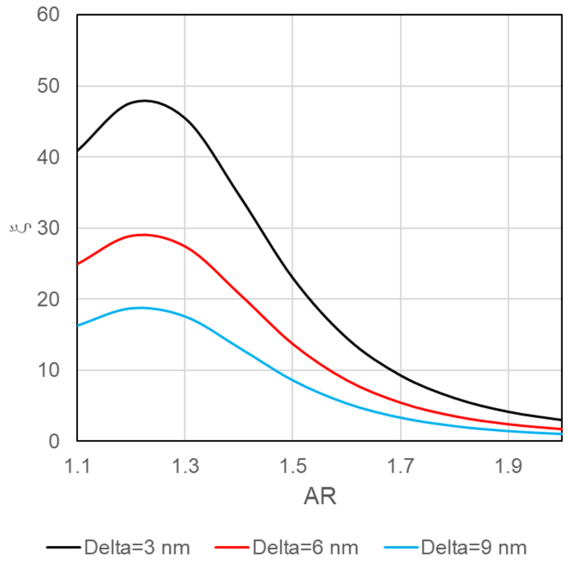

(**a**)

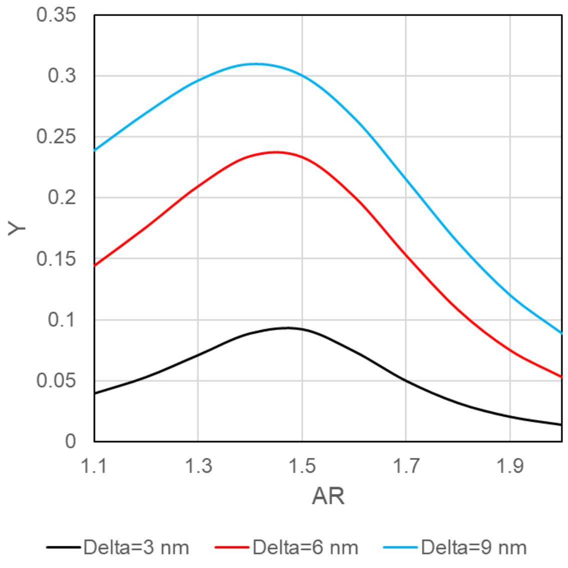

(**b**)

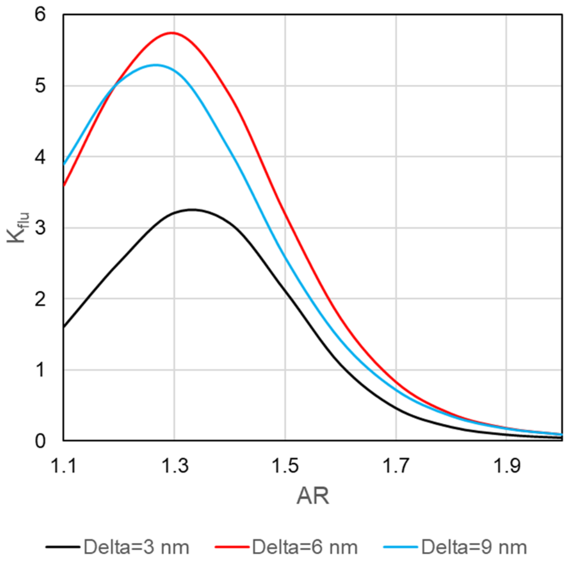

(**c**)

Figure S3. Dependence of: (**a**) – field enhancement coefficient ξ, (**b**) quantum yield Y, (**c**) – fluorescence enhancement coefficient K_{flu} vs AR; TM, "AuNR - TagRFP".

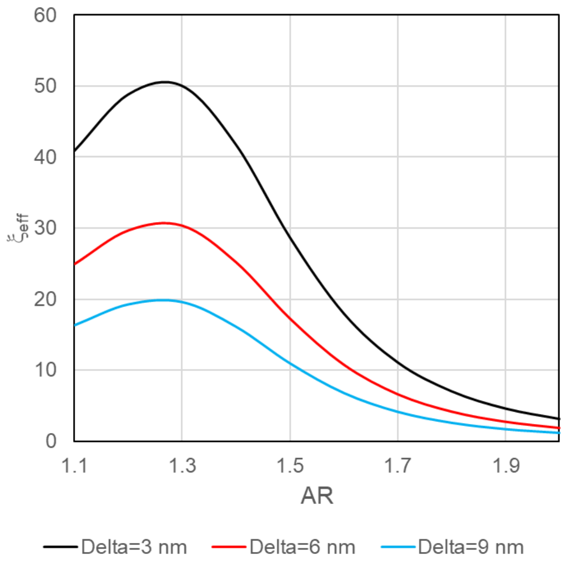

(**a**)

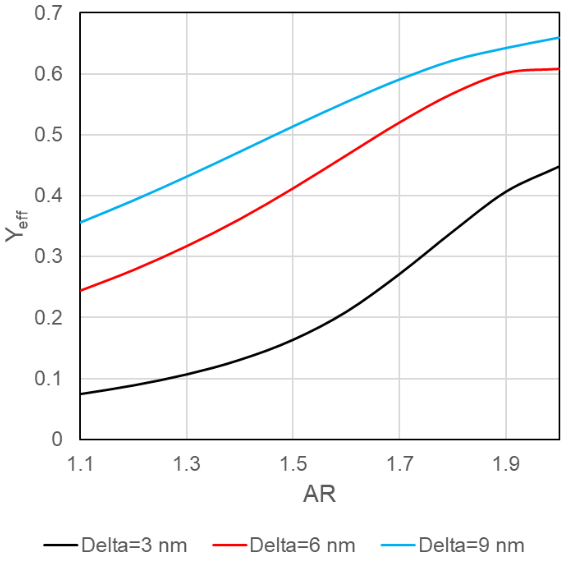

(**b**)

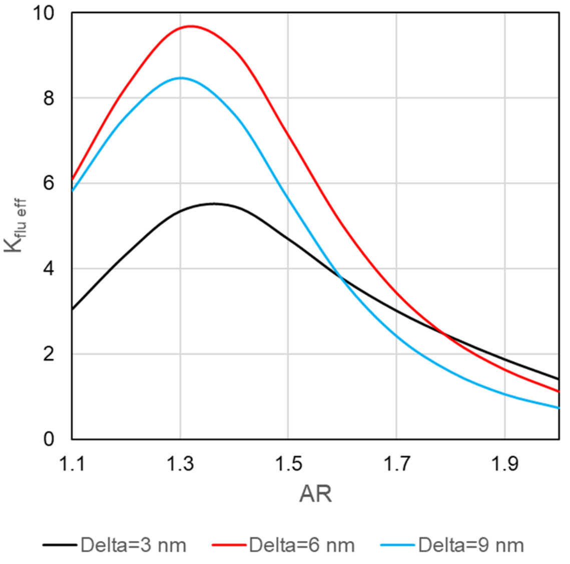

(**c**)

Figure S4. Dependence of: (**a**) – field enhancement coefficient ξ_{eff}, (**b**) quantum yield Y_{eff}, (**c**) – fluorescence enhancement coefficient $K_{flu\ eff}$ vs AR; RM, “AuNR - TagRFP”.
